# The effect of mahjong/bridge intellectual sports on the subjective wellbeing of middle-aged and older adults: an empirical analysis from the mixed cross-section data of CHARLS in China

**DOI:** 10.3389/fpubh.2025.1552180

**Published:** 2025-04-25

**Authors:** Qi-fei Xia, Guo-you Qin, Fei-long Yang, Zheng Li

**Affiliations:** ^1^School of Physical Education, Ankang College, Ankang, China; ^2^College of Physical Education, Hanjiang Normal University, Shiyan, China; ^3^Faculty of Education, Shenzhen University, Shenzhen, China

**Keywords:** intellectual sports, subjective wellbeing, social interaction, cognitive ability, healthy aging

## Abstract

**Background:**

As China’s population ages, intellectual sports have become a key leisure activity with a significant impact on the subjective wellbeing of middle-aged and older adult individuals. These activities promote social engagement, counteract the “empty nest” phenomenon, and offer a pathway to successful aging.

**Methods:**

This study utilized mixed cross-sectional data from the China Health and Retirement Longitudinal Study (CHARLS), encompassing 36,934 adults aged 45 and above. The relationship between intellectual sports, such as mahjong and bridge, and subjective wellbeing was analyzed using ordinal logistic regression modeling.

**Results:**

Participation in mahjong and bridge was positively associated with subjective wellbeing, a finding that remained consistent across multiple regression tests. Social interaction and cognitive ability emerged as critical mediators, enhancing wellbeing through increased social engagement and cognitive stimulation. Retirement status moderated this relationship, with retired individuals showing a stronger propensity to engage in these activities, thereby boosting their wellbeing. It is noteworthy that this positive impact was more pronounced among women and rural and eastern participants.

**Conclusion:**

This study elucidates the mechanisms through which mahjong and bridge influence the subjective wellbeing of middle-aged and older adult Chinese individuals. The findings offer valuable insights for policymakers, suggesting that promoting diverse cultural and sports activities within older adult communities could foster socialization, prevent cognitive decline, and ultimately enhance wellbeing.

## 1 Introduction

According to the World Health Organization (WHO), the global population of individuals over 60 is rapidly increasing, projected to double to 2.1 billion by 2025 (1). In China, the world’s most populous developing country, the aging population is expanding even more quickly, with those aged 60 and over expected to comprise 28% of the population by 2040 (2). This accelerated demographic shift will place increasing demands on healthcare resources, potentially compromising the quality of life for older adults and exacerbating challenges within the public healthcare system. Given these demographic changes, the wellbeing of middle-aged and older adults warrants close examination. The “Opinions of the Central Committee of the Communist Party of China and the State Council on Strengthening the Work of the Elderly in the New Era” emphasize the need to enhance the sense of fulfillment, happiness, and security among the older adult (3). Subjective wellbeing has thus become a crucial indicator of quality of life, alongside economic and social progress (4–6). As China’s population ages, the pursuit of wellbeing has entered a new phase, reflecting more comprehensive and elevated standards of living. Enhancing the wellbeing of the middle-aged and older adult has become a key concern in China’s modernization and development efforts. The 14th Five-Year Plan for the Development of the National Aging Career and Pension Service System explicitly encourages the development of sports and fitness programs tailored to older adults, including platforms for related competitions and demonstrations, in line with the concept of active aging (7). One such initiative is “mind sports,” a term popularized by the International Federation of Mind Sports in 2005 to describe intellectual games aimed at developing cognitive abilities. In China, intellectual sports like mahjong, bridge, and chess have been integrated into community life and are particularly popular among middle-aged and older adult individuals. Studies indicate that these activities not only reduce the risk of dementia (8) and alleviate anxiety (9, 10) but also promote social interaction (11), enrich leisure time (12), support active aging (13), and potentially enhance subjective wellbeing. Numerous studies have explored the impact of physical exercise on the wellbeing of the older adult, such as fitness qigong (2), air volleyball (14), and walking (15), all of which have been shown to significantly improve subjective wellbeing. However, the role of intellectual sports in enhancing wellbeing remains underexplored, particularly in comparison to these physical activities. Chinese society, with its rich history and cultural heritage, has long embraced traditional intellectual games such as mahjong and card games. These activities, with their diverse regional rules and standards, have become integral to the leisure lives of many, especially among the middle-aged and older adult. While mahjong, bridge, and other intellectual sports are popular in China and other developing countries, there is a lack of clear research on their impact on subjective wellbeing. Few studies have examined intellectual exercise as a core factor influencing the wellbeing of older adults, and the potential mechanisms at play remain underexplored. This study, utilizing data from the China Health and Retirement Longitudinal Study (CHARLS), aims to address this gap by using ordered logistic regression to investigate the relationship between intellectual sports, such as mahjong and bridge, and the subjective wellbeing of middle-aged and older adult people. The study also seeks to analyze the possible mechanisms involved. The main contributions of this study are threefold. First, while intellectual sports like Sudoku, solitaire, and checkers have been shown to affect health and wellbeing in developed countries, there is a scarcity of large-scale surveys extending these findings to other regions. This research, focused on China, the world’s largest developing country, synthesizes data from several significant surveys to explore whether these activities influence wellbeing, thereby providing essential evidence and practical solutions for older adult service centers, nursing care institutions, and gerontological education centers in China. Second, the study delves into the mechanisms and regulatory factors underlying the relationship between intellectual sports and wellbeing, utilizing a variety of tests to ensure robust results. It also considers the heterogeneity of subjective wellbeing among middle-aged and older adult individuals, accounting for factors such as gender culture and the urban–rural divide, to offer more contextually relevant insights. Finally, as China faces the challenges of an aging society, issues such as lack of childcare, low social participation, and spiritual emptiness may exacerbate the difficulties associated with “empty-nest” syndrome, affecting both the physical and mental health of older adults (16). By exploring the impact of intellectual sports on subjective wellbeing, this study not only promotes social participation and a sense of happiness in old age but also provides targeted policy recommendations to address the multifaceted needs of China’s aging population.

## 2 Literature review

### 2.1 Explanation of the connotation of subjective wellbeing

China’s economic development and technological reforms have significantly improved living conditions, shifting the public’s pursuit of subjective wellbeing from mere survival to higher-quality goals focused on enjoyment and development. Subjective wellbeing, a key indicator of mental health and quality of life, has long been a topic of scholarly interest in Chinese society. Historically, Confucianism and Taoism have shaped perspectives on wellbeing, with Confucian thought emphasizing the transformation of sorrow into joy and Taoism advocating concern for collective happiness (17). These philosophical traditions reflect a deep-rooted aspiration for a better life. As society progresses, the desire for happiness has intensified, drawing the attention of numerous scholars across disciplines. Subjective wellbeing is now explored from various perspectives. Psychologists commonly define it as the balance of positive and negative emotions, where greater positive emotions lead to higher happiness (18). Sociologists, however, view subjective wellbeing from a developmental perspective, encompassing both the pleasure individuals derive from life and the sense of value achieved through self-actualization (19). It represents an individual’s overall assessment of their environment and societal functioning, reflecting life satisfaction and value attribution (20). In 1974, economist Richard Easterlin introduced the “Easterlin Paradox,” prompting economists to re-evaluate the pursuit of happiness (21). Some scholars, from the perspectives of happiness economics and hedonism, assess subjective wellbeing through subjective evaluations and positive emotional responses to consumption activities (22). Today, self-assessed subjective wellbeing is commonly used in sociological and economic research as a proxy for evaluating happiness (23). Theoretical frameworks provide rich analytical tools for studying subjective wellbeing. Self-discrepancy theory (24) suggests that cognitive biases between one’s real, ideal, and perceived selves influence emotional experiences, with positive emotions enhancing psychological functioning and wellbeing (25). Goal content theory further emphasizes that subjective wellbeing is closely tied to intrinsic needs and internal drives, with happiness increasing when these needs are met or goals are achieved (26, 27).

In summary, subjective wellbeing can be measured through multidimensional or unidimensional approaches, with various disciplines exploring its core connotations, measurement methods, and constituent dimensions. Life satisfaction, a cognitive dimension of wellbeing (28), is a subjective evaluation of an individual’s quality of life based on personal standards (29) and is commonly used to measure subjective wellbeing (30, 31). Due to differences in living environments, life experiences, and self-worth, individuals lack a unified standard for evaluating subjective wellbeing, which can fluctuate with subjective and objective influences. Building on previous studies (32), this research focuses on life satisfaction as the primary variable to comprehensively analyze trends in subjective wellbeing among middle-aged and older adult individuals, providing robust support for empirical research.

### 2.2 Mahjong/bridge intellectual sports and subjective wellbeing

The World Mind Games, initiated by the International Mind Sports Alliance (IMSA), integrates board and card games like Mahjong, Bridge, and Chess into an international sporting event, highlighting their social value through diversity, innovation, and popularization. Mahjong and other intellectual sports are particularly popular among the older adult in China, serving as a social and entertaining activity that requires hand-eye coordination, mental agility, and interaction (33). Research shows that middle-aged and older adult individuals who regularly engage in social activities like visiting friends, playing mahjong, or participating in sports clubs can effectively alleviate depressive symptoms (34). Longitudinal studies indicate that loneliness increases with age, but regular participation in leisure activities, such as mahjong or card games, helps maintain social connections, reducing loneliness and enhancing wellbeing (35). Psychosocial adaptation during aging is crucial for promoting healthy aging. Persistence theory (36) and continuity theory (37) suggest that older adults achieve greater satisfaction by maintaining continuity with their younger selves. Engaging in enjoyable and meaningful activities, such as square dancing, mahjong, or chess, fosters positive social adaptation and better integration into social groups (38). Activity theory further emphasizes the link between activity and wellbeing (39). Older adults who maintain active social participation and positive social relationships tend to experience better health and wellbeing in later life (40). As middle-aged and older adult individuals transition from occupational to leisure roles, activities like playing mahjong or cards become essential for spending time, relieving stress, and fostering interests, hobbies, and skills, thereby enriching their spiritual lives. Based on this, the following hypotheses are proposed:

*Hypothesis H1*: Participation in mahjong/bridge intellectual sports significantly contributes to the subjective wellbeing of middle-aged and older adult people.

### 2.3 Mechanisms related to the effect of mahjong/bridge intellectual sports on subjective wellbeing of middle-aged and older adult people

Mahjong and bridge, as intellectual sports, possess both social and recreational attributes that significantly impact the subjective wellbeing of middle-aged and older adult individuals. Participation in these activities not only enhances social interactions and cognitive abilities but also serves as a key mechanism influencing wellbeing. Social interaction, in particular, plays a crucial role. Defined as interactions between individuals or groups under certain conditions (41), social interaction is essential for emotional and social wellbeing. Harvard University researchers found that, regardless of age, playing mahjong fosters emotional connections, expands social networks, and stimulates the release of oxytocin and endorphins, promoting health and comfort (42). Social interaction theory and social exchange theory suggest that individuals exist within a network of social relationships, where social interaction involves exchanging rewards and punishments (43, 44). In the context of Chinese society, the motivation for middle-aged and older adult people to participate in intellectual sports like mahjong or bridge may stem less from a desire for social engagement and more from the pursuit of material gain through winning, which in turn shapes social relationships. Moreover, the benefits of social interaction on subjective wellbeing are increasingly evident. Leisure activities with friends are recognized as important social resources (45), and the trust, mutual support, and respect that emerge from deep emotional bonds can enhance the wellbeing of older adults (46). Studies have also found that older individuals with close social networks report higher happiness scores (47). Improved interpersonal skills and relationships lead to greater social capital, embedding individuals within a social network that fosters a stronger sense of identity and value (48). Cognitive ability may also serve as a key mechanism (49). Engaging in intellectually demanding activities like playing mahjong or cards requires coordination of various cognitive abilities, including attention, observation, memory, language, and communication (50). Numerous studies have shown that intellectual sports such as mahjong and bridge enhance cognitive performance in middle-aged and older adults (51–53). Frequent participation in these games not only involves upper extremity physical activity but also provides rich emotional and environmental stimuli, which may increase cerebral blood flow, stimulate the central nervous system, improve brain metabolism, and promote adaptive changes in brain structure and function (34, 54, 55). However, evidence linking social activities to cognitive functioning remains limited, as highlighted by a randomized controlled trial (56). Theoretically, social activities like mahjong may stimulate cognitive reserve, potentially supporting cognitive outcomes and promoting healthy aging in middle-aged and older adults (57, 58). This protective effect against cognitive decline (59) is significant, as cognitive deterioration can impair quality of life and limit independence (60). A longitudinal study in the UK found a link between cognitive performance and positive wellbeing in older adults, with exercise mitigating the adverse effects of lower wellbeing on cognitive function (61). Older adults with higher cognitive abilities exhibit greater emotional resilience, showing smaller decreases in positive emotions and smaller increases in negative emotions when exposed to stressors (62). Therefore, it is hypothesized that participation in intellectual sports like mahjong or card games can protect cognitive abilities in middle-aged and older adults, leading to higher levels of wellbeing. Based on this, the following hypothesis is proposed:

*Hypothesis H2*: Participation in mahjong/bridge intellectual sports can enhance subjective wellbeing by promoting social interaction among middle-aged and older adult people.

*Hypothesis H3*: Participation in mahjong/bridge intellectual sports can enhance subjective wellbeing by improving the cognitive ability of middle-aged and older adult people.

### 2.4 Moderating effects of retirement

In addition to exploring the mechanisms described, it is crucial to examine the potential moderating effects of retirement among middle-aged and older adults. Retirement marks a significant transition, influencing work status, social networks, income, and lifestyle (63). Retirement systems differ between China, which primarily enforces mandatory retirement, and the West, where voluntary or flexible retirement is more common. This distinction affects how middle-aged and older adult individuals utilize their free time and experience aging (64). Grossman’s study suggests that lower income post-retirement encourages a shift from health behaviors to social activities, such as playing mahjong and poker (65). With increased free time and a slower pace of life, retirees may seek leisure activities like dancing, playing cards, chess, or attending senior centers to enrich their lives and enhance life satisfaction (66). However, as the global retirement age rises, the opportunity for such leisure activities may diminish, potentially reducing subjective wellbeing. A study in Spain using a dynamic rational expectations model found that delaying retirement by 2 years increases labor supply and reduces pension costs but results in significant welfare losses (67). Based on this, the following hypothesis is proposed:

*Hypothesis H4*: Retirement plays a moderating role in the effect of participation in mahjong/bridge intellectual sports on subjective wellbeing of middle-aged and older adults.

**Figure 1:**
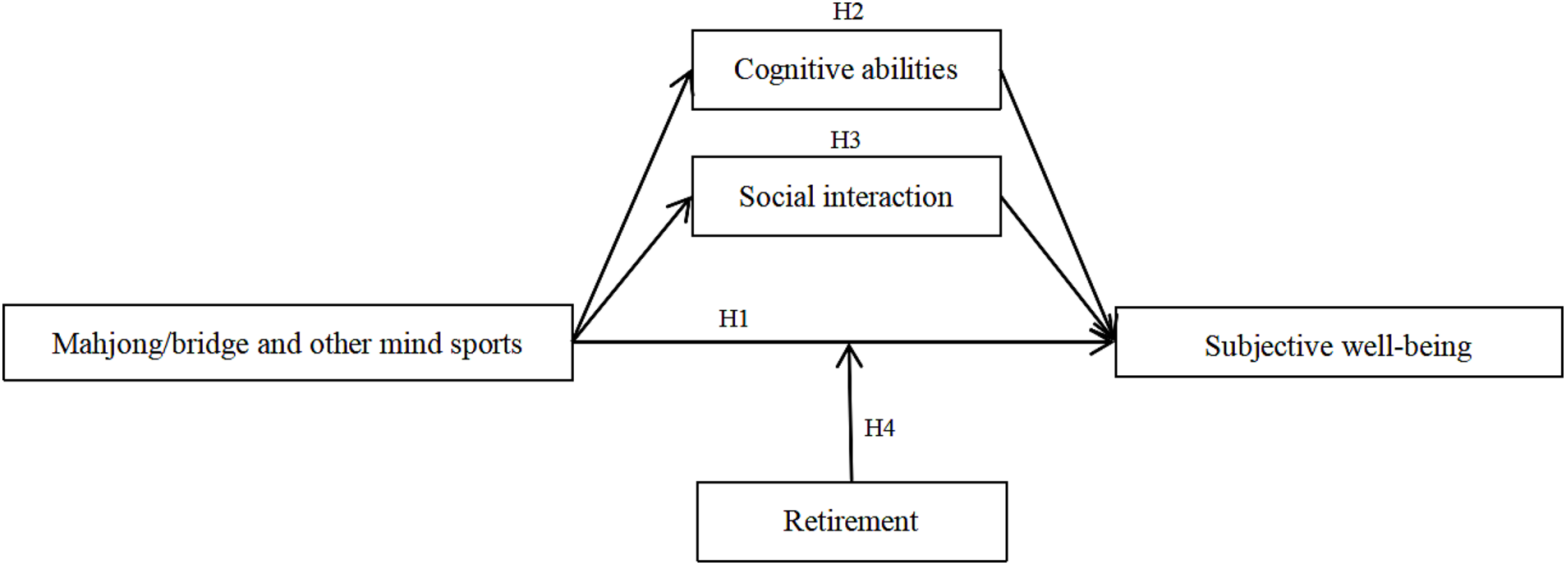
Theoretical framework.

Through the theoretical analysis and hypothesis derivation above, the theoretical model of participation in mahjong/bridge intellectual sports and subjective wellbeing of middle-aged and older adult people was constructed to quantitatively assess and analyze the causal relationship and mechanism of action. See Figure 1.

## 3 Materials and methods

### 3.1 Data sources

The data for this study were primarily sourced from the China Health and Retirement Longitudinal Study (CHARLS), which aims to provide high-quality microdata representing households and individuals aged 45 and above in China. The CHARLS surveys, conducted in 2011, 2013, 2015, and 2018, focus on issues related to population aging, including health, work, and retirement. The surveys cover 150 county-level units, 450 village-level units, and approximately 17,000 individuals across 10,000 households, ensuring good national representation. Because the 2020 data collection coincided with the peak of the epidemic, the survey methodology was replaced by partial telephone interviews for face-to-face interviews, resulting in a significant decline in respondent participation, and the impact of the epidemic itself on health behaviors and psychological status may have obscured trend analysis, this study used data from the four-period CHARLS mixed cross-section survey, and the 2020 data were subjected to a robustness test, with data retained only for people aged 45 years and older Middle-aged and older adult survey sample. After excluding cases with missing values and outliers, we obtained 36,934 valid samples: 8,196 from 2011, 9,555 from 2013, 10,123 from 2015, 9,060 from 2018, 12,002 from 2020.

### 3.2 Variable selection

#### 3.2.1 Dependent variable

The dependent variable is subjective wellbeing. Life satisfaction is a stable measure of people’s long-term wellbeing based on the sum of individual respondents’ multidimensional evaluations of life (68). Therefore, the life satisfaction index in the CHARLS questionnaire was used to measure subjective wellbeing, with the question “In general, are you satisfied with your life?.” The question was “In general, are you satisfied with your life?,” and the answers, “not at all satisfied, not too satisfied, relatively satisfied, very satisfied, extremely satisfied,” were assigned values from 1 to 5, meaning that the higher the value, the higher the subjective happiness of the middle-aged and older adult.

#### 3.2.2 Independent variable

The independent variable is mahjong/bridge intellectual sports, etc. The CHARLS questionnaire question is “Have you done any of the following activities in the past month - playing mahjong, chess, cards, etc.,” and the answer yes is assigned a value of 1, and no is assigned a value of 0, making it a dichotomous variable.

#### 3.2.3 Mediating variables

The mediator variables were social interaction and cognitive functioning. Social interaction was measured using the CHARLS questionnaire item, “Have you done any of the following activities in the past month—visiting, socializing with friends?” This was coded as a dichotomous variable (yes = 1, no = 0). Cognitive ability, following Lei’s framework (69), included memory and mental condition. Memory was assessed through immediate and delayed recall, ranging from 0 to 10. Mental status comprised time orientation, calculation ability, and graph recognition and drawing, with a total score of 11. The combined cognitive ability score (0–21) was used, with higher values indicating better cognitive function in middle-aged and older adult individuals.

#### 3.2.4 Moderator variables

The moderating variable is the retirement status of middle-aged and older adults. The CHARLS questionnaire is titled “Have you taken retirement (including early retirement) or retired internally?” Respondents answered yes with a value of 1, otherwise with a value of 0, a dichotomous variable.

#### 3.2.5 Control variables

Control variables included the basic characteristics of the middle-aged and older adults: gender (female = 0, male = 1); age (year of residence minus year of birth); household registration (rural = 0, urban = 1); marital status (not in marriage = 0, in marriage = 1); ethnicity (other ethnicity = 0, Han = 1); health status (very unhealthy = 1, relatively unhealthy = 2, average = 3, relatively healthy = 4, very healthy = 5); smoking (no = 0, yes = 1); alcohol consumption (no = 0, yes = 1); and region (West = 0, Central = 2, East = 3). Refer to Table 1.

**Table 1:** Descriptive statistics for each variable.

Variables	Full sample (*N* = 36,934)		Participation in mahjong/bridge mind games (*N* = 7,625)		No mahjong/bridge mind games (*N* = 29,309)	
	Mean	SD	Mean	SD	Mean	SD
Mahjong/bridge intellectual sports	0.206	0.405	1	0	0	0
SWB	3.220	0.589	3.334	0.496	3.190	0.607
Social interaction	0.369	0.482	0.570	0.495	0.316	0.464
Cognitive ability	12.354	3.327	13.274	2.865	12.114	3.396
Retirement	0.151	0.358	0.198	0.399	0.138	0.345
Gender	0.519	0.500	0.604	0.488	0.496	0.499
Age	59.294	8.820	58.617	8.536	59.469	8.884
Marital status	0.903	0.296	0.921	0.269	0.898	0.302
Household registration	0.761	0.426	0.690	0.462	0.779	0.414
Ethnicity	0.934	0.249	0.961	0.191	0.926	0.261
Health status	3.097	0.930	3.166	0.882	3.079	0.940
Smoking	0.447	0.497	0.551	0.497	0.420	0.493
Alcohol consumption	0.480	0.500	0.556	0.496	0.459	0.498
Region	2.031	0.815	2.050	0.769	2.026	0.826

### 3.3 Research methods

Based on the research hypotheses, data processing and statistical analysis were conducted using STATA 17.0. Given that the explanatory variable, subjective wellbeing, is an ordered categorical variable, an Ologit regression analysis (ordered logistic Steele model) was employed to better capture the dominant impact effect. The benchmark model was specified as follows:


(1) $$SWB_{i,t}=\beta_{0}+\beta_{1}MS_{i,t}+\beta_{2-5}\sum \mathrm{Control}+\delta_{i,t}$$

In equation 1, Where *i* stands for sample individual *i*, *t* stands for time, *SWB* stands for individual subjective wellbeing, *MS* stands for mahjong/bridge intellectual sports, Control stands for a series of related control variables such as gender, household registration, etc., *β*_1_ to *β*_5_ are the regression coefficients of each variable, and *δ* is the residual term. If based on the assumptions made in this study, *β*_1_ is significantly positive or negative.

In order to test the two important mechanisms of action of social interaction and cognitive ability, the stepwise regression method proposed by Baron and Kenny (70) was borrowed to initially test whether there is a mediating effect in the above research hypotheses. The setup model is as follows:


(2) $$SWB_{i}=\alpha_{0}+\alpha_{1}MS_{i}+\alpha_{x}\mathrm{Contro}l_{i}+\delta_{i}$$

(3) $$\mathrm{Mediatio}n_{i}=\beta_{0}+\beta_{1}MS_{i}+\beta_{x}\mathrm{Contro}l_{i}+\delta_{i}$$

(4) $$SWB_{i}=\gamma_{0}+\gamma_{1}MS_{i}+\gamma_{2}\mathrm{Mediatio}n_{i}+\gamma_{x}\mathrm{Contro}l_{i}+\delta_{i}$$

In Equation 1, the model *MS* variable is intellectual sports, including playing mahjong, cards, chess, etc., *SWB_i_* is the probability of sports participation, *Control_i_* is the relevant control variable, and *α*_1_ is the parameter to be estimated. Equations 2, 3 variable interpretation is the same as Equation 1, and *Mediation_i_* is the mediating variable social interaction, cognitive ability (Equation 4). The regression model is used to test whether the core independent variables have a significant effect on the dependent variable, and if it is significant, the core independent variables are further estimated whether the core independent variables have a significant effect on each of the mediating variables, and finally the core independent variables and the mediating variables are jointly estimated in the regression, and if the mediation mechanism is established, it is necessary to satisfy the existence of significance at the same time of *α*_1_, *β*_1_, and *γ*_2_. When *γ*_1_ is not significant then it is a full mediating effect, on the contrary, if *γ*_1_ is significant and *γ*_1_ < *α*_1_, then it is a partial mediating effect. However, there are some problems that cannot be ignored; the method does not fully focus on the share of the mediating effect in the total effect, its efficacy is low, and the estimation of the effect may be biased. In order to more accurately express the role played by the mediating variables, Sobel test was used to test the reliability of the results obtained from the mediating effects, and the KHB decomposition method developed by Karlson et al. (71) was also used to analyze the significance and percentage of the model’s direct effects, indirect effects and total effects.

In order to test whether there is a moderating effect of retirement, model (1) was established with subjective wellbeing as the dependent variable, intellectual sports such as mahjong/bridge as the independent variable, and retirement as the moderating variable to verify whether the main effect hypothesis is valid. The hierarchical regression model is as follows:


(5) $$SWB_{1}=\alpha_{0}+\sum \alpha_{i}MS_{i}+\sum \alpha_{j}X_{j}\sum \alpha_{k}X_{k}+\delta_{i}$$

In Equation 5, *SWB* is subjective wellbeing, *MS* is mahjong/bridge intellectual sports, *X_j_* is retirement moderating variable, *X_k_* is series of control variables such as gender, household registration, etc., and *δ* is random interference term. On this basis, the interaction term between intellectual sports such as mahjong/bridge and retirement is constructed, and model (2) is established to verify whether the moderating effect of retirement is valid. The hierarchical regression model is as follows:


(6) $$SWB_{2}=\alpha_{0}+\sum \alpha_{i}MS_{i}+\sum \alpha_{j}X_{j}+\sum \alpha_{k}X_{k}+\sum \alpha_{i}X_{i}\sum \alpha_{j}X_{j}+\delta_{i}$$

In Equation 6, *SWB*_2_ represents the dependent variable subjective wellbeing, *MS_i_* is the independent variable mahjong/bridge and other intellectual sports, *X_j_* is the retirement moderator variable, *X_k_* is a series of control variables such as gender, household registration, etc., *∑ α_i_ MS_i_ ∑ α_j_ X_j_* refers to the interaction term of the independent and moderator variables, and *δ* is a random disturbance term.

## 4 Empirical results

### 4.1 Main characteristics of the survey sample

To better understand the variability in the baseline survey of middle-aged and older adults over recent years, we categorized the dependent, independent, mediating, moderating, and control variables by year. Table 2 shows that subjective wellbeing is highest in terms of comparative satisfaction (66.53%), with stability observed between comparative satisfaction and extreme satisfaction from 2011 to 2018. Intellectual sports participation, such as mahjong or bridge, remains low, with 79.36% of participants not engaging and no significant trend over time. Social interaction is reported by 36.87% of the older adult, peaking in 2013 at 40.66%, then declining annually. Cognitive ability, measured as a continuous variable, averaged 12.354 across the sample, indicating a relatively low level. For moderating variables, 84.90% of older adults are retired or retired early, with a gradual decline in the proportion of non-retirees from 2011 to 2018. We also observed that there were a large number of missing values for alcohol consumption in the 2020 data, so they were not included in the study. The distribution of each variable among middle-aged and older adults showed overall stratified differences due to the 2020 epidemic.

**Table 2:** Statistics on the main characteristics of middle-aged and older adult people.

Categories		Master Sample	2011	2013	2015	2018	2020
Totally		36,934 (100%)	8,196 (22.19%)	9,555 (25.87%)	10,123 (27.41%)	9,060 (24.53%)	12,002 (100%)
SWB	Not at all satisfied	285 (0.77%)	71 (0.87%)	80 (0.84%)	56 (0.55%)	78 (0.86%)	225 (1.87%)
	Not too satisfied	1,920 (5.2%)	625 (7.63%)	552 (5.78%)	388 (3.83%)	355 (3.92%)	808 (6.73%)
	Relatively satisfied	24,572 (66.53%)	5,893 (71.9%)	6,872 (71.92%)	5,909 (58.37%)	5,898 (65.10%)	6,937 (57.79%)
	Very satisfied	9,696 (26.25%)	1,563 (19.07%)	1,977 (20.69%)	3,580 (35.37%)	2,576 (28.43%)	3,475 (28.95%)
	Extremely satisfied	461 (1.25%)	44 (0.54%)	74 (0.77%)	190 (1.88%)	153 (1.69%)	558 (4.65%)
Mahjong/Bridge Intellectual Sports	No	29,309	6,639	7,403	7,928	7,339	9,996
		(79.36%)	(81%)	(77.48%)	(78.32%)	(81%)	(83.28%)
	Yes	7,625	1,557	2,152	2,195	1,721	2,007
		(20.64%)	(19%)	(22.52%)	(21.68%)	(19%)	(16.72%)
Gender	Female	17,764 (48.1%%)	4,050 (49.41%)	4,702 (49.21%)	4,837 (47.78%)	4,175 (46.08%)	5,803 (48.35%)
	Male	19,170 (51.90%)	4,853 (50.79%)	3,432 (47.03%)	5,286 (52.22%)	4,885 (53.92%)	6,200 (51.65%)
Age	Continuous variable						
Marital status	Not in marriage	3,589 (9.72%)	738 (9%)	878 (9.19%)	1,019 (10.07%)	954 (10.53%)	1,415 (11.79%)
	Marriage	33,345 (90.28%)	7,458 (91%)	8,677 (90.81%)	9,104 (85.93%)	8,106 (89.47%)	10,588 (88.21%)
Household registration	Rural	8,814 (23.86%)	1,765 (21.53%)	2,188 (22.90%)	2,329 (23.01%)	2,532 (27.95%)	5,103 (42.51%)
	Urban	28,120 (76.14%)	6,431 (78.47%)	7,367 (77.10%)	7,794 (76.99%)	6,528 (72.05%)	6,900 (57.49%)
Ethnicity	Other ethnicity	2,456 (6.65%)	572 (6.98%)	629 (6.58%)	648 (6.40%)	607 (6.70%)	732 (6.10%)
	Han	34,478 (93.35%)	7,624 (93.02%)	8,926 (93.42%)	9,475 (93.60%)	8,453 (93.30%)	11,271 (93.90%)
Health status	Very unhealthy	1,396 (3.78%)	261 (3.18%)	368 (3.85%)	361 (3.57%)	406 (4.48%)	690 (5.75%)
	Relatively unhealthy	6,416 (17.37%)	1,627 (19.85%)	1,608 (16.83%)	1,600 (15.81%)	1,581 (17.45%)	1,960 (16.33%)
	Average	20,063 (54.32%)	4,300 (52.46%)	5,326 (55.74%)	5,641 (55.72%)	47.96 (52.94%)	6,331 (52.75%)
	Relatively healthy	5,325 (14.42%)	1,493 (18.22%)	1,386 (14.51%)	1,257 (12.42%)	1,189 (13.12%)	1,547 (12.89%)
	Very healthy	3,734 (10.11%)	515 (6.28%)	867 (9.07%)	1,264 (12.49%)	1,088 (12.01%)	1,475 (12.29%)
Smoking	No	20,416 (55.28%)	4,810 (58.69%)	5,352 (56.01%)	5,437 (53.71%)	4,817 (53.17%)	6,597 (54.96%)
	Yes	16,518 (44.72%)	3,386 (41.31%)	4,203 (43.99%)	4,686 (46.29%)	4,243 (46.83%)	5,406 (45.04%)
Alcohol consumption	No	19,223	4,764	4,978	5,151	4,330	
		(52.05%)	(58.13%)	(52.10%)	(50.88%)	(47.79%)	
	Yes	17,711	3,432	4,577	4,972	4,730	
		(47.95%)	(41.87%)	(47.90%)	(49.12%)	(52.21%)	
Region	West	11,696	2,563	3,076	3,260	2,797	4,548
		(31.67%)	(31.27%)	(32.19%)	(32.20%)	(30.87%)	(37.89%)
	Central	12,384	2,756	3,150	3,332	3,146	3,072
		(33.53%)	(33.63%)	(32.97%)	(32.92%)	(34.72%)	(25.59%)
	East	12,854	2,877	3,329	3,531	3,117	4,383
		(34.80%)	(35.10%)	(34.84%)	(34.88%)	(34.40%)	(36.52%)
Social interaction	No	23,318	5,337	5,670	6,426	5,885	7,843
		(63.13%)	(65.12%)	(59.34%)	(63.48%)	(64.96%)	(65.34%)
	Yes	13,616	2,859	3,885	3,697	3,175	4,160
		(36.87%)	(34.88%)	(40.66%)	(36.52%)	(35.04%)	(34.66%)
Cognitive ability	Continuous variable						
Retirement	No	31,358	7,179	8,202	8,646	7,331	9,588
		(84.90%)	(87.59%)	(85.84%)	(85.41%)	(80.92%)	(79.89%)
	Yes	5,576	1,017	1,353	1,477	1,729	2,414
		(15.10%)	(12.41%)	(14.16%)	(14.59%)	(19.08%)	(20.11%)

### 4.2 Baseline regression results

**Figure 2:**
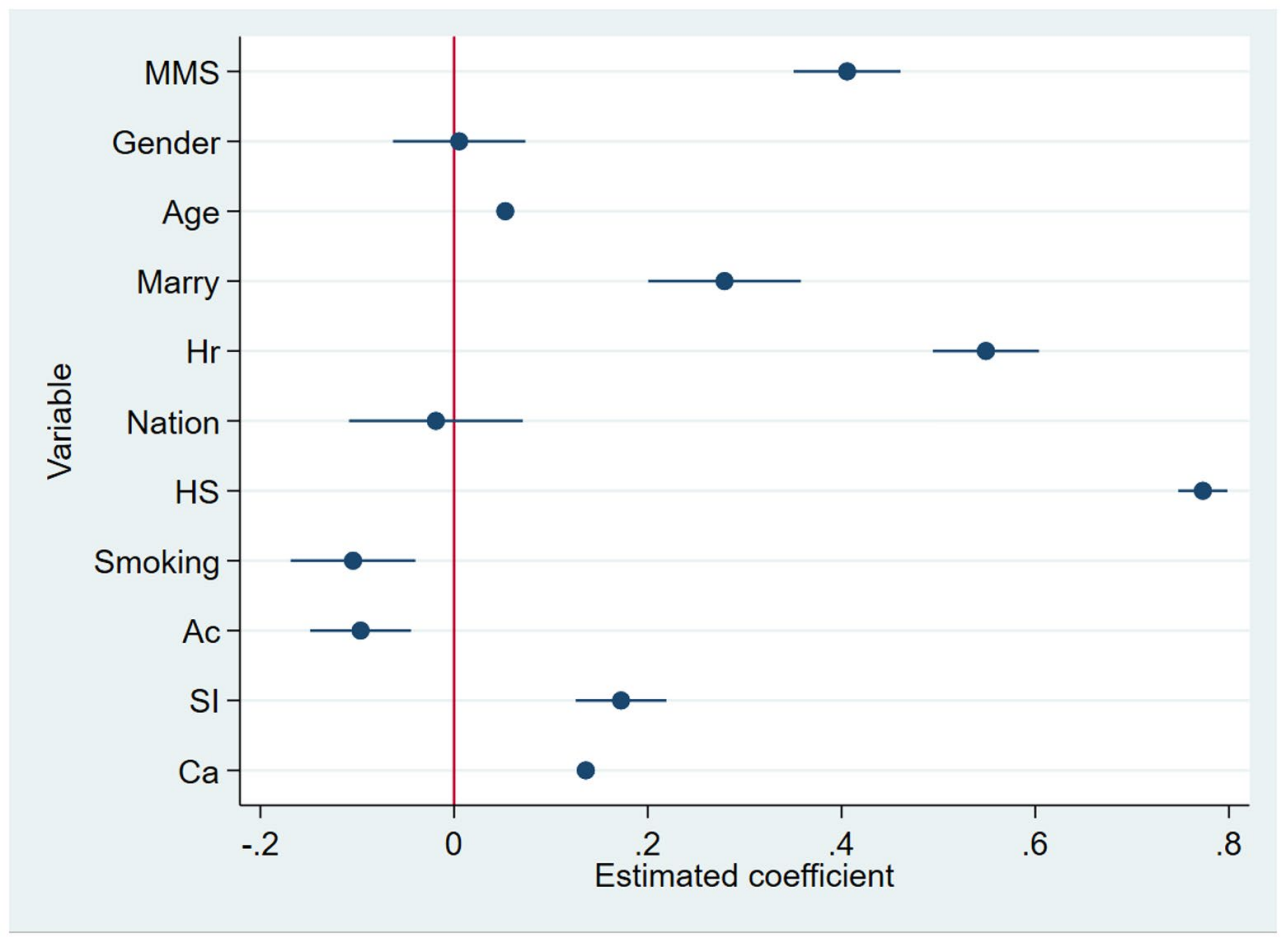
Visual analysis of regression results.

Table 3 presents the benchmark regression results. In model (1), participation in mahjong/bridge intellectual sports positively predicts the subjective wellbeing of middle-aged and older adult individuals, with a significant regression coefficient of 0.452 (*p* < 0.01) and an odds ratio (OR) of 1.571. This indicates a 57.1% higher probability of enhanced wellbeing for participants compared to non-participants. Model (2), which includes control variables, shows that the predictive effect remains significant (*β* = 0.523, *p* < 0.01) with an OR of 1.686, suggesting a 68.6% higher probability of improved wellbeing. The model’s explanatory power increased, with *R*^2^ rising from 0.004 to 0.075. Control variable coefficients indicate higher wellbeing among males, older adults, married individuals, urban residents, those in good health, non-smokers, non-drinkers, and residents of the eastern region, while ethnicity showed no significance. Models (3) to (6), covering sub-year samples from 2011 to 2018, confirm the consistent positive impact of mahjong/bridge on wellbeing over time, all significant at the 1% level. Models (7) and (8), which include mediating variables, reveal that social interaction and cognitive ability positively influence wellbeing, both significant at the 1% level. In model (9), the overall *R*^2^ increases to 0.096, further enhancing the model’s explanatory power while maintaining the significance of the findings. To enhance the clarity of the regression results for each variable, we visualized the data using the Coefplot command in Stata 17.0. This approach effectively illustrates the advantages of the dependent variables and presents the estimated coefficients along with confidence intervals, facilitating significance assessment. Figure 2 displays the standardized regression distributions, which align closely with the model’s estimation results.

**Table 3:** Regression results of mahjong/bridge intellectual sports on subjective wellbeing of middle-aged and older adult people.

	(1)	(2)	(3)	(4)	(5)	(6)	(7)	(8)	(9)
Variables	SWB	SWB	2011	2013	2015	2018	SWB	SWB	SWB
Mahjong/bridge intellectual sports	0.452***	0.523***	0.615***	0.626***	0.422***	0.493***	0.473***	0.444***	0.405***
	(0.0259)	(0.0273)	(0.0619)	(0.0550)	(0.0497)	(0.0562)	(0.0278)	(0.0276)	(0.0282)
Gender		0.129***	0.213***	0.140**	0.193***	0.104	0.144***	−0.00893	0.00423
		(0.0344)	(0.0724)	(0.0705)	(0.0656)	(0.0706)	(0.0344)	(0.0348)	(0.0349)
Age		0.0368***	0.0376***	0.0364***	0.0231***	0.0413***	0.0377***	0.0525***	0.0530***
		(0.00135)	(0.00308)	(0.00283)	(0.00243)	(0.00277)	(0.00136)	(0.00144)	(0.00144)
Marital status		0.337***	0.346***	0.240***	0.344***	0.383***	0.348***	0.270***	0.280***
		(0.0400)	(0.0910)	(0.0839)	(0.0729)	(0.0776)	(0.0400)	(0.0402)	(0.0402)
Household registration		0.249***	0.172***	0.150***	0.274***	0.398***	0.257***	0.549***	0.552***
		(0.0265)	(0.0602)	(0.0552)	(0.0493)	(0.0511)	(0.0265)	(0.0280)	(0.0280)
Ethnicity		−0.0158	0.112	−0.0713	0.0659	−0.179**	−0.00683	−0.0131	−0.00606
		(0.0462)	(0.0995)	(0.0956)	(0.0868)	(0.0909)	(0.0462)	(0.0463)	(0.0464)
Health status		0.786***	0.770***	0.811***	0.792***	0.755***	0.784***	0.777***	0.776***
		(0.0129)	(0.0300)	(0.0270)	(0.0238)	(0.0250)	(0.0129)	(0.0130)	(0.0131)
Smoking		−0.144***	−0.243***	−0.119*	−0.148**	−0.192***	−0.145***	−0.102***	−0.104***
		(0.0326)	(0.0691)	(0.0669)	(0.0619)	(0.0667)	(0.0326)	(0.0329)	(0.0329)
Alcohol consumption		−0.0822***	−0.0369	−0.191***	−0.124**	−0.0970*	−0.0916***	−0.0902***	−0.0977***
		(0.0264)	(0.0596)	(0.0548)	(0.0483)	(0.0524)	(0.0264)	(0.0266)	(0.0266)
Region		0.0340**	0.00147	0.0406	0.0283	0.0721**	0.0304**	−0.0221	−0.0243*
		(0.0141)	(0.0311)	(0.0290)	(0.0258)	(0.0286)	(0.0141)	(0.0143)	(0.0143)
Social interaction							0.217***		0.173***
							(0.0237)		(0.0239)
Cognitive ability								0.138***	0.137***
								(0.00391)	(0.00391)
Pseudo *R*^2^	0.004	0.075	0.067	0.077	0.075	0.078	0.076	0.095	0.096
Prob > chi2	0.000	0.000	0.000	0.000	0.000	0.000	0.000	0.000	0.000
Observations	36,934	36,934	8,196	9,555	10,123	9,060	36,934	36,934	36,934

The parentheses are standard errors, ***, **, and * indicate significance at 1, 5, and 10% levels, respectively.

### 4.3 Robustness testing

**Figure 3:**
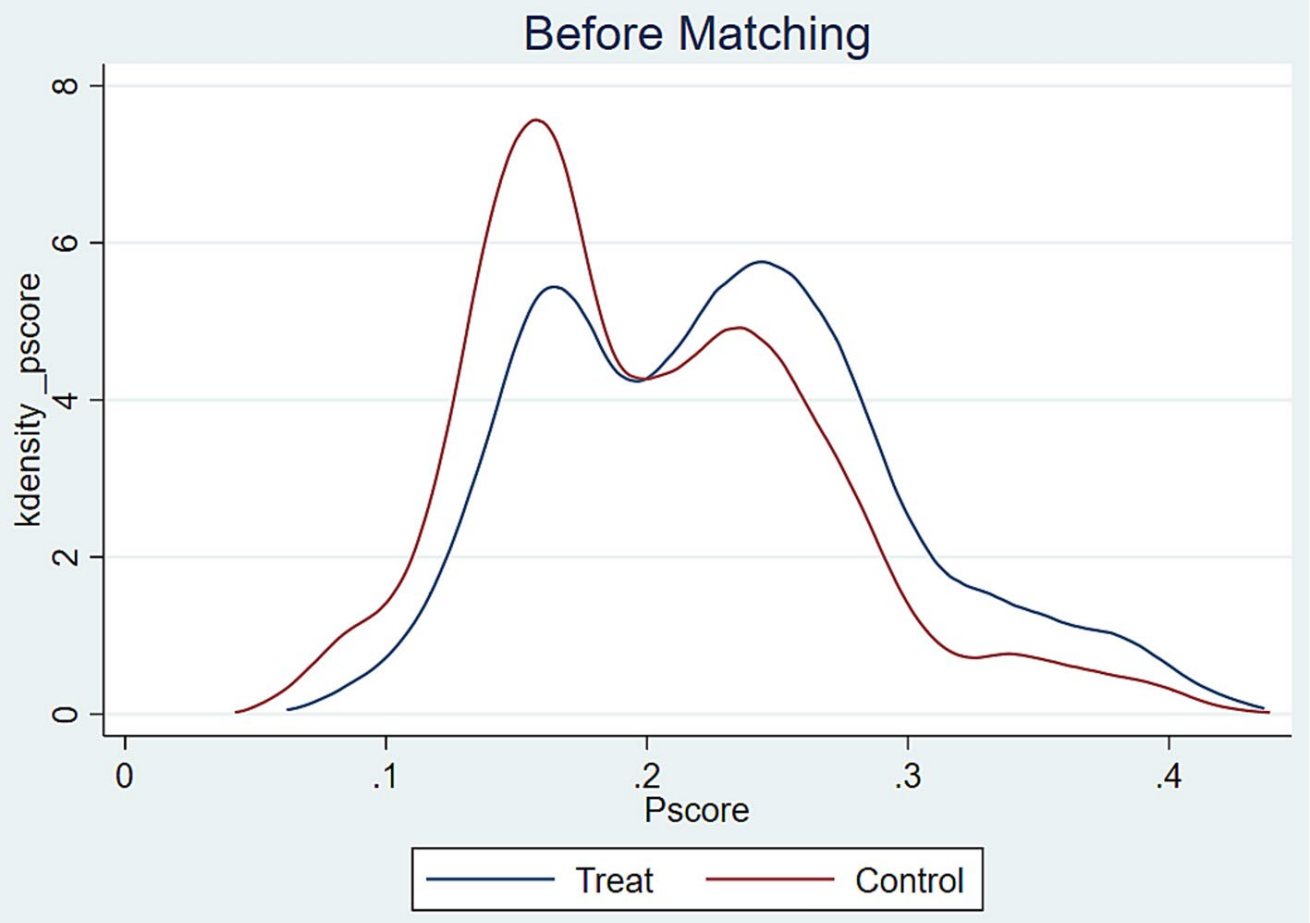
Kernel density function plot before PSM matching.

**Figure 4:**
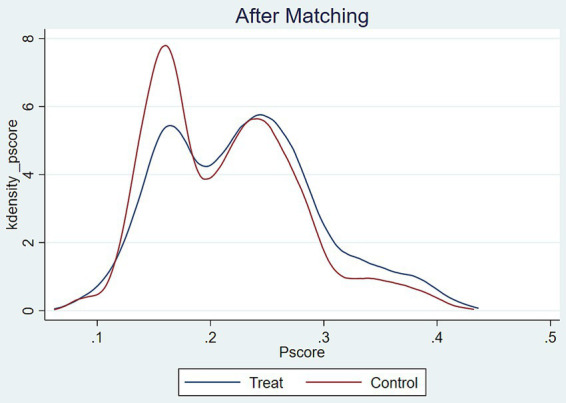
Kernel density function plot after PSM matching.

In order to ensure the heterogeneity and stability of the results of the test of the effect of intellectual sports such as mahjong/bridge on the subjective wellbeing of middle-aged and older adult people, four ways will be taken to conduct the robustness test. (1) Supplementary empirical data. 2020 fifth-round survey data were collected at the beginning of the New Crown epidemic outbreak, which made middle-aged and older adult people unavoidably feel panicked and overwhelmed at the beginning of public health emergencies. Therefore, we only subjected the 2020 data to robustness tests, and found that intellectual sports such as mahjong/bridge still had a significant effect on the subjective wellbeing of middle-aged and older adult people in model (1), suggesting that participation in intellectual sports such as home-based mahjong/bridge could eliminate epidemic prevention anxiety to a certain extent during the quarantine period of the epidemic, thus enhancing subjective wellbeing. (2) Replacing the empirical model. A multiple linear regression model (OLS model) was established to test its robustness, and the results showed that the effect of intellectual sports such as mahjong/bridge on the subjective wellbeing of middle-aged and older adult people was significantly positive, which verified the reliability of the results. (3) Change the dependent variable. The regression analysis of the dependent variable subjective wellbeing, with “quite satisfied, very satisfied and extremely satisfied” classified as 1 and “not at all satisfied and not very satisfied” classified as 0, found that the coefficient of the impact effect was still significantly positive, confirming the robustness of the results (Table 4). (4) Propensity score matching. To address potential selection bias in the regression analysis of the effects of mahjong/bridge on the subjective wellbeing of middle-aged and older adult individuals, we employed Propensity Score Matching (PSM). Given that PSM requires dichotomous variables, we calculated the average treatment effect using K-nearest neighbor matching (1:4), radius matching, and kernel matching, with subjective wellbeing as the dependent variable. The PSM results showed that the T-values for the average treatment effect (ATT) exceeded the critical value (Table 5). Additionally, PSM kernel density plots demonstrated a significant reduction in mean line distance post-matching, suggesting reduced selection bias and consistent results (Figures 3, 4).

**Table 4:** Robustness test results.

Variables	(1)	(2)	(3)
	2020	Replacement of empirical models	Changing the dependent variable
Mahjong/bridge intellectual sports	0.265***	0.140***	0.0617***
	(0.0474)	(0.00720)	(0.00554)
Pseudo *R*^2^	0.039	0.122	0.094
Observations	12,002	36,934	36,934

The parentheses are standard errors, ***, **, and * indicate significance at 1, 5, and 10% levels, respectively.

**Table 5:** Results of propensity score matching.

Matching method	Process group	Control group	ATT	SE	T-stat
Prematch	3.334	3.190	0.143	0.007	19.06***
After matching					
Nearest neighbor matching	3.334	3.204	0.129	0.008	16.20***
Nuclear matching	3.334	3.194	0.139	0.006	20.47***
Radius matching (0.01)	3.334	3.195	0.138	0.006	20.18***

The parentheses are standard errors, ***, **, and * indicate significance at 1, 5, and 10% levels, respectively.

### 4.4 Mechanism of action analysis

#### 4.4.1 Stepwise regression test

Following the mediation effect test procedure, we conducted preliminary tests using stepwise regression to examine the mediation effects of social interaction and cognitive ability. The results indicate that both social interaction and cognitive ability significantly and positively influence the subjective wellbeing of middle-aged and older adult individuals, with significance at the 1% level. Notably, the main effect coefficients in models (3) and (5) are lower than in model (1), suggesting the presence of an indirect effect mediated by social interaction and cognitive ability. (See Table 6).

**Table 6:** Stepwise regression test results for social interaction, cognitive ability.

Variables	(1)	(2)	(3)	(4)	(5)
	SWB	Social interaction	SWB	Cognitive ability	SWB
Mahjong/bridge intellectual sports	0.523***	1.055***	0.473***	0.417***	0.444***
	(0.0273)	(0.0269)	(0.0278)	(0.0225)	(0.0276)
Social interaction			0.217***		
			(0.0237)		
Cognitive ability					0.138***
					(0.00391)
Pseudo *R*^2^	0.075	0.043	0.076	0.035	0.095
Observations	36,934	36,934	36,934	36,934	36,934

The parentheses are standard errors, ***, **, and * indicate significance at 1, 5, and 10% levels, respectively.

#### 4.4.2 Tests for mediating effects

Building on the initial stepwise regression analysis, we further investigated the mechanisms of social interaction and cognitive ability using KHB decomposition, Sobel test, and Bootstrap methods. The results, presented in Table 6, indicate that 10.33 and 18.98% of the effects of middle-aged and older adult participation in mahjong/bridge intellectual sports on subjective wellbeing can be attributed to social interaction and cognitive ability, respectively, with both mediating effects being significant. The Sobel test results also support this, with *Z*-values greater than 1.96 and *p*-values below 0.01, confirming the significance of these mediating effects. Additionally, the Bootstrap method showed that the 95% confidence intervals for both direct and indirect effects did not include zero, further affirming that social interaction and cognitive ability partially mediate the relationship between mahjong/bridge intellectual sports and subjective wellbeing (See Table 7).

**Table 7:** Mediating effects of social interaction and cognitive ability.

	Social interaction			Cognitive ability		
	Coefficient	Lower limit	Upper limit	Coefficient	Lower limit	Upper limit
Total effect	0.527***	0.474	0.581	0.547***	0.493	0.601
Direct effect	0.473***	0.418	0.527	0.443***	0.389	0.498
Indirect effect	0.054***	0.042	0.066	0.103***	0.092	0.115
Percentage of contribution		10.33%			18.98%	

Sobel test		
Sobel	0.016***	0.027***
Goodman-1 (Aroian)	0.016***	0.027***
Goodman-2	0.016***	0.027***
*Z*	10.44	17.32

Bootstrap Results – 1000reps		
Indirect effect (95% CI)	[0.013, 0.019]	[0.024, 0.029]
Direct effect (95% CI)	[0.110, 0.135]	[0.100, 0.125]
*p*-value	0.000***	0.000***

The parentheses are standard errors, ***, **, and * indicate significance at 1, 5, and 10% levels, respectively.

### 4.5 Moderating effects test

To assess the moderating effect of retirement on the relationship between mahjong and bridge intellectual sports and subjective wellbeing, interaction terms were constructed. Model (1) shows that retirement positively predicts subjective wellbeing among middle-aged and older adult individuals (*β* = 0.131, *p* < 0.01), indicating that retired individuals tend to experience higher subjective wellbeing. In Model (2), the interaction term between mahjong/bridge participation and retirement was significant (*β* = 0.202, *p* < 0.01), suggesting that retirement enhances the positive effect of mahjong/bridge intellectual sports on subjective wellbeing. Thus, retirement acts as a moderator in this relationship (See Table 8).

**Table 8:** Results of the moderating effect of retirement.

Variables	(1)	(2)
	SWB	SWB
Mahjong/bridge intellectual sports	0.521***	0.483***
	(0.0273)	(0.0302)
Retirement	0.131***	0.0741*
	(0.0403)	(0.0447)
Mahjong/bridge intellectual sports × Retirement		0.202***
		(0.0687)
Pseudo *R*^2^	0.075	0.075
Observations	36,934	36,934

The parentheses are standard errors, ***, **, and * indicate significance at 1, 5, and 10% levels, respectively.

### 4.6 Heterogeneity analysis

There may be some differences in the effects of intellectual sports such as mahjong/bridge on the subjective wellbeing of middle-aged and older adult people with different characteristics, and this paper examines the heterogeneity of demographic characteristics of the middle-aged and older adult groups. The results showed that for different gender groups, intellectual sports such as mahjong/bridge had a significant positive effect on subjective wellbeing for both females and males. Therefore, the difference in coefficients between groups was tested by the method of seemingly uncorrelated model (SUEST), and the *p*-value of the coefficient of difference between groups was less than 0.05, which indicates that intellectual sports such as mahjong/bridge have a significantly higher effect on subjective wellbeing in females than in males. In terms of domicile, intellectual sports such as mahjong/bridge had a significant positive effect on the subjective wellbeing of both rural and urban middle-aged and older adult people. The coefficient of difference between groups in the seemingly unrelated model was significant (*p* < 0.01), indicating that intellectual sports such as mahjong/bridge have a greater effect on the subjective wellbeing of rural middle-aged and older adult people. See Table 9. According to the World Health Organization’s criteria for classifying the age of the older adult, 45–59 years old is the low old age, 60–74 years old is the middle old age, and 75 years old and above is the high old age (72), which was used as a categorical variable for the regression. The results show that intellectual sports such as mahjong/bridge have a significant positive effect on the subjective wellbeing of middle-aged and older adult people in different age groups, and the coefficient of variation between groups has a *p*-value greater than 0.05, so there is no significant between-group difference in this effect. According to the geographic division of China, the regression of regional samples was conducted separately, and it was found that mahjong/bridge and other intellectual sports had a significant positive effect on the subjective wellbeing of middle-aged and older adult people in different regions, but the effect was greater in the eastern region (*p*-value of the regression coefficient test for the two–two subgroups was 0.044, 0.790, 0.017, respectively). As far as marriage is concerned, the effect of intellectual sports such as mahjong/bridge on the subjective wellbeing of the older adult not in marriage is more obvious, and the coefficients of the core explanatory variables of the two groups of samples tweed are not significantly different (*p* = 0.253). See Table 10.

**Table 9:** Heterogeneity test regression results for gender and household registration.

Variables	(1)	(2)	(3)	(4)
	Female	Male	Rural	Urban
Mahjong/bridge intellectual sports	0.589***	0.479***	0.703***	0.473***
	(0.0417)	(0.0363)	(0.0546)	(0.0318)
Pseudo *R*^2^	0.073	0.076	0.092	0.072
Observations *p*	17,764	19,170 0.024	8,814	28,120 0.000

The parentheses are standard errors, ***, **, and * indicate significance at 1, 5, and 10% levels, respectively.

**Table 10:** Regression results of heterogeneity tests for age, region and marriage.

Variables	(5)	(6)	(7)	(8)	(9)	(10)	(11)	(12)
	Lower age	Middle old age	High old age	Western	Central	East	Not in marriage	marriage
Mahjong/bridge intellectual sports	0.497***	0.506***	0.651***	0.551***	0.429***	0.568***	0.607***	0.511***
	(0.0366)	(0.0432)	(0.123)	(0.0519)	(0.0441)	(0.0471)	(0.0941)	(0.0285)
Pseudo *R*^2^	0.070	0.070	0.061	0.064	0.077	0.079	0.091	0.073
Observations	19,804	15,011	2,119	11,696	12,384	12,854	3,589	33,345
*p* = 0.869, 0.182, 0.215				*p* = 0.044, 0.790, 0.017			*p* = 0.253	

The parentheses are standard errors, ***, **, and * indicate significance at 1, 5, and 10% levels, respectively.

## 5 Discussion

Using data from the China Health and Elderly Tracking Mixed Cross-Sectional Survey, we investigated the association between intellectual sports such as mahjong and bridge and subjective wellbeing among middle-aged and older adult populations. This study provides empirical insights for enhancing subjective wellbeing in these age groups, both within China and potentially in other regions. Our analysis also examines the underlying mechanisms linking these activities to wellbeing and evaluates how this relationship varies under different retirement scenarios, contributing to the existing body of research. Our results indicate that 66.53% of Chinese middle-aged and older adult individuals report a relatively high level of subjective wellbeing, aligning closely with the findings of Sun (73) and Zhao et al. (74). Compared to low-and middle-income countries like India and Viet Nam, China benefits from more developed eldercare infrastructure, public welfare and cultural programs, and social welfare subsidies (75, 76), which support a comfortable life in later years. However, disparities in happiness levels are observed when compared to European countries (77), likely influenced by regional cultural and economic factors. Among the control variables—gender, age, marital status, household registration, health status, smoking, alcohol consumption, and region—all showed significant effects on subjective wellbeing, except for ethnicity. This may be due to the substantial socio-economic advancements in ethnic minority regions in China, which have improved both material conditions and cultural life, contributing to a heightened sense of wellbeing. Notably, some ethnic minorities, such as the Uighurs and Hui, report higher happiness levels than the Han Chinese (78). These findings suggest that the development of a strong national community since China’s reform and opening up has played a critical role in fostering a sense of wellbeing.

We employed an ordered logistic (Ologit) regression model to assess the independent impact of mahjong and bridge on the subjective wellbeing of middle-aged and older adult individuals. Our analysis revealed that these intellectual sports significantly enhance subjective wellbeing, consistent with previous studies (79, 80). Given China’s accelerated aging and the increasing number of empty nesters, the financial burden on healthcare and public health services has grown (81). Promoting participation in leisure activities such as square dancing and mahjong can improve health outcomes and contribute to healthy aging (82). A cohort study of older adults in Japan found a significant association between leisure activities and lower mortality rates, with varying effects across different activities (83). In China, intellectual games like mahjong and poker are culturally significant and competitive. Middle-aged and older adult individuals often engage in these activities collectively, enjoying the mental stimulation and social interaction they provide, which significantly enhances their sense of wellbeing. The benefits of these games are multifaceted: physiologically, regular participation in group social activities like mahjong and bridge improves mental health, reduces depression risk, and counteracts age-related brain atrophy (84). Psychologically, these games involve complex rules that enhance hand-eye coordination and provide positive emotional experiences (79). Group intellectual sports such as mahjong, cards, and chess can effectively reduce loneliness, foster a positive self-concept, and offer social support, thereby significantly enhancing subjective wellbeing (8).

As middle-aged and older adult individuals face declining physical functions, increased comorbidity risks, and diminishing social roles, they are prone to negative emotional experiences, which can weaken their subjective wellbeing. Engaging in intellectual sports like mahjong and bridge not only fills leisure time and enriches life but also provides mental exercise, promotes social interaction, and improves overall life satisfaction. However, it is important to note that prolonged engagement in these activities can lead to sedentary behavior, increasing the risk of chronic diseases. For instance, excessive time spent on mahjong has been linked to negative effects on cardiovascular and digestive systems (12). Therefore, middle-aged and older adult individuals should regulate their participation in these intellectual sports, maintain moderation, and adopt a healthy lifestyle to prevent addiction and associated health risks. This study validated the partial mediating role of social interaction and cognitive ability, confirming that participation in intellectual sports such as mahjong and bridge enhances subjective wellbeing through two key pathways: fostering social interaction and improving cognitive ability. On one hand, intellectual sports like mahjong and bridge, which require minimal external resources and can be played in various settings, facilitate group activities that boost interpersonal connections and social support, thereby increasing wellbeing (85). The social bonds formed through these activities provide middle-aged and older adult participants with a greater sense of community and enjoyment.

On the other hand, evidence suggests that older adults who regularly engage in card games or mahjong demonstrate better performance in attention, long-term memory, and logical reasoning (86). Cognitive function, as a protective factor, contributes to more positive emotions and a stronger sense of wellbeing (62). Consequently, participation in these intellectual sports not only expands social networks and enhances emotional connections but also promotes hand-eye coordination, maintains memory and cognitive skills, reduces the risk of depression, and improves overall wellbeing. These findings offer policy recommendations for relevant government departments, emphasizing the importance of promoting moderate participation in intellectual sports like mahjong or solitaire through community outreach and health education. Additionally, policies should be developed to support the health and social activities of the older adult, including the provision of accessible venues, financial support, and the design of barrier-free spaces that accommodate the diverse cultural backgrounds of the older adult population. Retirement moderates the relationship between participation in mahjong and bridge intellectual sports and subjective wellbeing. Our data indicate that deeply engaged retired older adults experience a positive effect on subjective wellbeing (87). With more leisure time, retired individuals often choose activities like mahjong and card games to meet personal needs and enhance life satisfaction. According to activity theory, older adults require active engagement in various roles to maintain vitality (88). The shift from work to retirement prompts seniors to cultivate interests and hobbies, integrate into social life, and fulfill growing emotional needs, thereby enhancing their sense of belonging and wellbeing. This underscores the importance of providing diverse and personalized recreational activities in communities to promote social participation and improve wellbeing among retired individuals. Our study found that participation in mahjong and bridge intellectual sports had the greatest impact on enhancing subjective wellbeing among female and rural middle-aged and older adult individuals. In Chinese family culture, women often bear the responsibility for childcare and household tasks, leading them to prefer more static leisure activities like mahjong and meditation to promote health and relieve stress (89). In contrast, older men tend to engage in competitive activities such as brisk walking and tai chi. In rural China, where local sentiment and clan concepts are strong, group activities like playing mahjong and chess are common forms of entertainment for the older adult, fostering social capital and improving subjective wellbeing. Regionally, the development of China’s social security system for the older adult is characterized by a regional imbalance of “high in the east and low in the west” (90), with the rise in the level of socialized pensions in the eastern region giving more and more middle-aged and older adult people more leisure time to participate in the intellectual activities of mahjong and an easier time gaining a sense of satisfaction. Although the study found that there was no significant difference between the coefficients of age and marriage between groups. However, it is worth exploring that, as age continues to grow, the effect of recreational activities such as mahjong/bridge is conducive to meeting the spiritual needs of the middle-aged and older adult, but still needs to be avoided as a kind of gambling tool to spend the rest of their lives in the wilderness. Meanwhile, the absence of marriage may bring about the shrinkage of social network and more dependence on family support, while participation in intellectual activities such as mahjong can make individuals gain pleasure and positive emotions, which makes them more optimistic to face the difficulties and pressures of life.

However, this study has several limitations. First, the data were primarily collected through self-reports, which may introduce bias due to the Hawthorne effect. Future research should incorporate interviews and other methods to enhance data accuracy. Second, our study focused only on whether middle-aged and older adults participate in intellectual sports, without analyzing the frequency and intensity of participation, which should be addressed in subsequent studies. Third, while we explored the relationship between playing intellectual sports and subjective wellbeing, various confounding factors, such as community environment and public service management, were not measured. These should be considered in future research. Lastly, to gain a broader understanding of intellectual activity participation among middle-aged and older adult individuals, future studies should include cross-sectional comparisons using multi-country data, examining differences in subjective wellbeing across diverse social backgrounds, racial groups, and geographic regions.

## 6 Conclusion

Using cross-sectional data from the China Health and Elderly Tracking Survey, this study investigates the impact of intellectual sports such as mahjong and bridge on the subjective wellbeing of middle-aged and older adult Chinese individuals. We identify social interaction and cognitive ability as key mechanisms, and further explore the heterogeneous effects of retirement on these outcomes. Our findings reveal that participation in these activities significantly enhances subjective wellbeing, with results robust across different time points. Notably, social engagement and cognitive maintenance appear critical in mediating the positive effects of these intellectual sports. Middle-aged and older adult participants, particularly those in retirement, experience the greatest benefits. Additionally, Chinese women, rural areas and middle-aged and older adult people in the eastern region are more enthusiastic about playing mahjong, poker and other intellectual sports to spend leisure time together and gain a sense of wellbeing. These insights offer valuable directions for policymakers and healthcare providers in improving the health and quality of life for aging populations, thereby contributing to the broader goal of successful aging.

## Data Availability

Publicly available datasets were analyzed in this study. This data can be found at: https://charls.pku.edu.cn/en/.
