# Supplementary material for: The effect of mahjong/bridge intellectual sports on the subjective wellbeing of middle-aged and older adults: an empirical analysis from the mixed cross-section data of CHARLS in China
Source: Front Public Health. 2025 Apr 25;13:1552180. doi: 10.3389/fpubh.2025.1552180 (PMC12061696; doi:10.3389/fpubh.2025.1552180)
Supplement: Supplementary file 1 [file Data_Sheet_1.zip › Date set/Regression analysis.pdf]

Return to baseline：

```
. ologit swb social2

Iteration 0:   log likelihood = -32065.531
Iteration 1:   log likelihood = -31915.114
Iteration 2:   log likelihood = -31914.741
Iteration 3:   log likelihood = -31914.741

Ordered logistic regression                     Number of obs     =     36,934
                                                LR chi2(1)        =     301.58
                                                Prob > chi2       =     0.0000
Log likelihood = -31914.741                     Pseudo R2         =     0.0047
```

swb	Coef.	Std. Err.	z	P>|z|	[95% Conf.	Interval]
social2	.4520448	.0258738	17.47	0.000	.4013332	.5027565
/cut1	-4.777972	.0596118			-4.894809	-4.661135
/cut2	-2.673642	.0224046			-2.717554	-2.62973
/cut3	1.074626	.0133218			1.048516	1.100736
/cut4	4.482521	.0473929			4.389632	4.575409

Year-by-year regression analysis：

```
> 1 if year == 2011

Iteration 0:   log likelihood = -6709.6288
Iteration 1:   log likelihood = -6274.7913
Iteration 2:   log likelihood = -6255.6422
Iteration 3:   log likelihood = -6255.5693
Iteration 4:   log likelihood = -6255.5693

Ordered logistic regression                     Number of obs     =      8,196
                                                LR chi2(10)       =     908.12
                                                Prob > chi2       =     0.0000
Log likelihood = -6255.5693                     Pseudo R2         =     0.0677
```

swb	Coef.	Std. Err.	z	P>|z|	[95% Conf.	Interval]
social2	.6147589	.0618955	9.93	0.000	.4934458	.7360719
gender	.2130072	.072373	2.94	0.003	.0711587	.3548557
age	.03757	.0030796	12.20	0.000	.0315341	.043606
marry	.345616	.0910285	3.80	0.000	.1672034	.5240285
rural	.1715025	.060179	2.85	0.004	.0535538	.2894513
nation	.1117582	.0994904	1.12	0.261	-.0832394	.3067558
health	.769821	.0300258	25.64	0.000	.7109716	.8286705
smoke	-.2434093	.0691479	-3.52	0.000	-.3789367	-.107882
drinke	-.0368635	.0596358	-0.62	0.536	-.1537475	.0800205
[illegible]	[illegible]	[illegible]	[illegible]	[illegible]	[illegible]	[illegible]

```
. ologit swb social2 gender age marry rural nation health smoke drinke province1 if year == 2013

Iteration 0:   log likelihood =  -7696.029
Iteration 1:   log likelihood = -7124.8173
Iteration 2:   log likelihood = -7098.9137
Iteration 3:   log likelihood = -7098.8227
Iteration 4:   log likelihood = -7098.8227

Ordered logistic regression                     Number of obs     =      9,555
                                                LR chi2(10)       =    1194.41
                                                Prob > chi2       =     0.0000
Log likelihood = -7098.8227                     Pseudo R2         =     0.0776
```

swb	Coef.	Std. Err.	z	P>|z|	[95% Conf.	Interval]
social2	.6261074	.05496	11.39	0.000	.5183877	.7338271
gender	.1401617	.0704634	1.99	0.047	.0020559	.2782674
age	.0364075	.0028336	12.85	0.000	.0308538	.0419613
marry	.2401215	.0838991	2.86	0.004	.0756822	.4045608
rural	.1503066	.0552087	2.72	0.006	.0420994	.2585137
nation	-.0713245	.0956477	-0.75	0.456	-.2587905	.1161415
health	.8114865	.0269568	30.10	0.000	.7586522	.8643208
smoke	-.1191792	.0668573	-1.78	0.075	-.2502172	.0118587
drinke	-.1910448	.0547749	-3.49	0.000	-.2984017	-.0836879
[illegible]	[illegible]	[illegible]	[illegible]	[illegible]	[illegible]	[illegible]

```
. ologit swb social2 gender age marry rural nation health smoke drinke province1 if year == 2015

Iteration 0:   log likelihood = -9214.1156
Iteration 1:   log likelihood = -8526.7806
Iteration 2:   log likelihood = -8516.1765
Iteration 3:   log likelihood = -8516.1371
Iteration 4:   log likelihood = -8516.1371

Ordered logistic regression                     Number of obs     =     10,123
                                                LR chi2(10)       =    1395.96
                                                Prob > chi2       =     0.0000
Log likelihood = -8516.1371                     Pseudo R2         =     0.0758
```

swb	Coef.	Std. Err.	z	P>|z|	[95% Conf.	Interval]
social2	.4219347	.0496502	8.50	0.000	.3246222	.5192473
gender	.1928684	.065644	2.94	0.003	.0642086	.3215283
age	.0231473	.0024332	9.51	0.000	.0183784	.0279163
marry	.3436653	.0728626	4.72	0.000	.2008572	.4864735
rural	.2743132	.0493225	5.56	0.000	.177643	.3709835
nation	.0659181	.0868341	0.76	0.448	-.1042736	.2361098
health	.7919561	.0237598	33.33	0.000	.7453877	.8385244
smoke	-.1482355	.061895	-2.39	0.017	-.2695475	-.0269235
drinke	-.1236615	.0483044	-2.56	0.010	-.2183363	-.0289867
[illegible]	[illegible]	[illegible]	[illegible]	[illegible]	[illegible]	[illegible]

```
. ologit swb social2 gender age marry rural nation health smoke drinke province1 if year == 2018

Iteration 0:   log likelihood = -7916.7388
Iteration 1:   log likelihood = -7314.0596
Iteration 2:   log likelihood = -7298.6836
Iteration 3:   log likelihood = -7298.6242
Iteration 4:   log likelihood = -7298.6242

Ordered logistic regression                     Number of obs     =      9,060
                                                LR chi2(10)       =    1236.23
                                                Prob > chi2       =     0.0000
Log likelihood = -7298.6242                     Pseudo R2         =     0.0781
```

swb	Coef.	Std. Err.	z	P>|z|	[95% Conf.	Interval]
social2	.4931095	.0562001	8.77	0.000	.3829593	.6032596
gender	.1039877	.0705679	1.47	0.141	-.0343229	.2422983
age	.0413301	.0027654	14.95	0.000	.0359101	.0467501
marry	.3830953	.0775942	4.94	0.000	.2310135	.5351771
rural	.3976091	.0510783	7.78	0.000	.2974974	.4977208
nation	-.1786804	.0909122	-1.97	0.049	-.356865	-.0004957
health	.7548557	.0250183	30.17	0.000	.7058208	.8038906
smoke	-.191692	.0667086	-2.87	0.004	-.3224384	-.0609455
drinke	-.0969502	.0524276	-1.85	0.064	-.1997063	.0058059
[illegible]	[illegible]	[illegible]	[illegible]	[illegible]	[illegible]	[illegible]

Addition to the analysis of mediating variables in social interactions：

```
. ologit swb social2 gender age marry rural nation health smoke drinke province1 social1

Iteration 0:   log likelihood = -32065.531
Iteration 1:   log likelihood = -29678.214
Iteration 2:   log likelihood = -29607.931
Iteration 3:   log likelihood = -29607.663
Iteration 4:   log likelihood = -29607.663

Ordered logistic regression                     Number of obs     =     36,934
                                                LR chi2(11)       =    4915.74
                                                Prob > chi2       =     0.0000
Log likelihood = -29607.663                     Pseudo R2         =     0.0767
```

swb	Coef.	Std. Err.	z	P>|z|	[95% Conf.	Interval]
social2	.473357	.0278386	17.00	0.000	.4187943	.5279197
gender	.1440947	.0344458	4.18	0.000	.0765821	.2116073
age	.0376809	.001358	27.75	0.000	.0350193	.0403425
marry	.3483872	.0400419	8.70	0.000	.2699065	.4268679
rural	.2574618	.0265251	9.71	0.000	.2054737	.30945
nation	-.0068316	.0462173	-0.15	0.882	-.0974159	.0837526
health	.7839603	.0129415	60.58	0.000	.7585955	.8093251
[illegible]	[illegible]	[illegible]	[illegible]	[illegible]	[illegible]	[illegible]

Addition of cognitive ability mediator variable analysis：

```
. ologit swb social2 gender age marry rural nation health smoke drinke province1 cognition

Iteration 0:   log likelihood = -32065.531
Iteration 1:   log likelihood = -29127.141
Iteration 2:   log likelihood = -29008.141
Iteration 3:   log likelihood = -29007.898
Iteration 4:   log likelihood = -29007.898

Ordered logistic regression                     Number of obs     =     36,934
                                                LR chi2(11)       =    6115.27
                                                Prob > chi2       =     0.0000
Log likelihood = -29007.898                     Pseudo R2         =     0.0954
```

swb	Coef.	Std. Err.	z	P>|z|	[95% Conf.	Interval]
social2	.4439072	.0276366	16.06	0.000	.3897404	.498074
gender	-.0089348	.0348427	-0.26	0.798	-.0772252	.0593557
age	.0524646	.0014388	36.46	0.000	.0496446	.0552846
marry	.2699788	.0401663	6.72	0.000	.1912544	.3487032
rural	.5485386	.0280258	19.57	0.000	.493609	.6034682
nation	-.0131162	.0463384	-0.28	0.777	-.1039378	.0777054
health	.7768749	.0130494	59.53	0.000	.7512986	.8024512
smoke	-.1024974	.0328783	-3.12	0.002	-.1669376	-.0380572
drinke	-.0902241	.0265624	-3.40	0.001	-.1422853	-.0381628
[illegible]	[illegible]	[illegible]	[illegible]	[illegible]	[illegible]	[illegible]

Total sample regression analysis:

```
. ologit swb social2 gender age marry rural nation health smoke drinke province1 social1 cognition

Iteration 0:   log likelihood = -32065.531
Iteration 1:   log likelihood = -29103.107
Iteration 2:   log likelihood = -28981.881
Iteration 3:   log likelihood = -28981.638
Iteration 4:   log likelihood = -28981.638

Ordered logistic regression                     Number of obs     =     36,934
                                                LR chi2(12)       =    6167.79
                                                Prob > chi2       =     0.0000
Log likelihood = -28981.638                     Pseudo R2         =     0.0962
```

swb	Coef.	Std. Err.	z	P>|z|	[95% Conf.	Interval]
social2	.4051065	.0281609	14.39	0.000	.3499122	.4603008
gender	.0042277	.0349058	0.12	0.904	-.0641864	.0726418
age	.0530025	.0014413	36.77	0.000	.0501776	.0558275
marry	.2798079	.0402	6.96	0.000	.2010173	.3585986
rural	.5521553	.028042	19.69	0.000	.4971941	.6071165
nation	-.0060551	.0463601	-0.13	0.896	-.0969193	.0848091
health	.7756216	.0130567	59.40	0.000	.750031	.8012122
smoke	-.1036216	.0328928	-3.15	0.002	-.1680903	-.039153
[illegible]	[illegible]	[illegible]	[illegible]	[illegible]	[illegible]	[illegible]
